# Seed selection strategy in global network alignment without destroying the entire structures of functional modules

**DOI:** 10.1186/1477-5956-10-S1-S16

**Published:** 2012-06-21

**Authors:** Bingbo Wang, Lin Gao

**Affiliations:** 1 School of Computer Science and Technology, Xidian University, 710071, China; 2 School of Computer Science and Technology, Xi'an University of Technology, 710048, China

## Abstract

**Background:**

Network alignment is one of the most common biological network comparison methods. Aligning protein-protein interaction (PPI) networks of different species is of great important to detect evolutionary conserved pathways or protein complexes across species through the identification of conserved interactions, and to improve our insight into biological systems. Global network alignment (GNA) problem is NP-complete, for which only heuristic methods have been proposed so far. Generally, the current GNA methods fall into global heuristic seed-and-extend approaches. These methods can not get the best overall consistent alignment between networks for the opinionated local seed. Furthermore These methods are lost in maximizing the number of aligned edges between two networks without considering the original structures of functional modules.

**Methods:**

We present a novel seed selection strategy for global network alignment by constructing the pairs of hub nodes of networks to be aligned into multiple seeds. Beginning from every hub seed and using the membership similarity of nodes to quantify to what extent the nodes can participate in functional modules associated with current seed topologically we align the networks by modules. By this way we can maintain the functional modules are not damaged during the heuristic alignment process. And our method is efficient in resolving the fatal problem of most conventional algorithms that the initialization selected seeds have a direct influence on the alignment result. The similarity measures between network nodes (e.g., proteins) include sequence similarity, centrality similarity, and dynamic membership similarity and our algorithm can be called Multiple Hubs-based Alignment (MHA).

**Results:**

When applying our seed selection strategy to several pairs of real PPI networks, it is observed that our method is working to strike a balance, extending the conserved interactions while maintaining the functional modules unchanged. In the case study, we assess the effectiveness of MHA on the alignment of the yeast and fly PPI networks. Our method outperforms state-of-the-art algorithms at detecting conserved functional modules and retrieves in particular 86% more conserved interactions than IsoRank.

**Conclusions:**

We believe that our seed selection strategy will lead us to obtain more topologically and biologically similar alignment result. And it can be used as the reference and complement of other heuristic methods to seek more meaningful alignment results.

## Background

Understanding complicated networks of interacting proteins is a major challenge in systems biology. Computational methods to analyze and compare networks are also being developed at a fast pace, resulting in an explosive growth in available protein-protein interaction (PPI) data. Comparative PPI network analysis across species has been used to understand similarities and differences between species at the systemic level [1,2], in particular, identify conserved protein subnetworks across species that are believed to represent evolutionarily conserved functional modules [3]. Generally, a PPI network is represented as an undirected graph in which nodes indicate proteins and edges indicate interactions. Comparing PPI networks usually translates into the form of network alignment to identify the pairs of homologous proteins from two different organisms. In the biological context, Network alignment has been proven to be a valuable tool for understanding the structure and function of complex biological networks in evolutionary and systems biology [1]. It also can be used to validate PPI conserved across multiple species and detect evolutionary conserved pathways or protein complexes [1,10]. The interactions are usually obtained by high-throughput experimental bio-techniques, the two most commonly used of which are yeast two-hybrid screening, resulting in binary interaction data [4-6], and protein complex purification methods using mass-spectrometry, resulting in co-complex data [7-9]. As suggested by increasing evidence, protein interaction modules that are conserved across species may exist.

Several methods have been proposed to perform local network alignment (LNA) of PPI networks [11-13]. LNA algorithms aim to find local small regions of isomorphism (same subnetworks) corresponding to pathways and protein complexes, which are conserved in PPI networks of different species. Such alignments can be ambiguous because a single protein in one network can be offered implausibly numerous different pairings in different local alignments.

A global network alignment (GNA), in contrast, provides a unique alignment from a protein in one network to only one protein in another network. The aim in GNA is to seek the best overall consistent alignment across all nodes simultaneously for the applications of functional ortholog identification. GNA has been studied previously in the context of biological networks [14,15]. Guided by the intuition that two nodes should be matched only if their neighbours can also be matched, IsoRank [14] aims to maximize the overall match between the two networks using a greedy strategy. The latest MI-GRAAL [15] can align networks of any type heavily relying on "graphlet degrees," which is a quantification of the topological similarity between nodes [15]. MI-GRAAL and IsoRank are all based on the seed-and-extend heuristic approach for solving the assignment problem.

Generally, the current GNA methods [14,15] fall into global heuristic seed-and-extend approach with two steps: (1) find a seed pair of nodes that can be aligned with the highest similarity score; (2) extend and align the neighbourhoods of the pair of seed nodes. The fatal drawback of this strategy is that the final alignment result largely depends on the selected seeds. These methods can not get the best overall consistent alignment between networks for the opinionated local seeds. Especially, when the PPI networks have a great way different size, the smaller network will be simply aligned to some local regions of the larger one.

**Figure 1:**
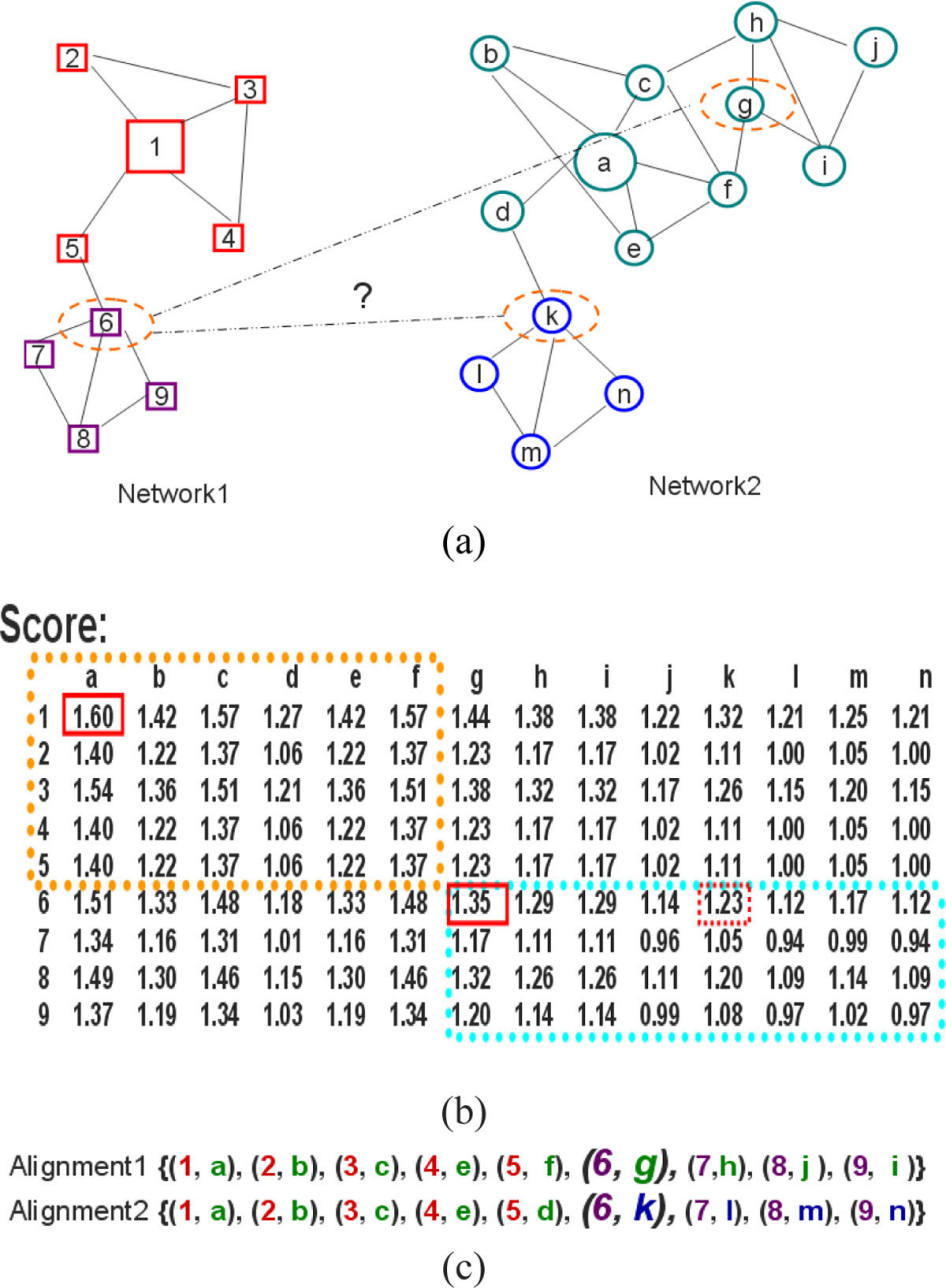
**A schematic example to show our motivation**. (a) Two Networks to be aligned. (b) Similarity scores between nodes of the two networks. (c) Two different alignment results.

In Figure 1 we give a schematic example to show this phenomenon and the intuition behind our method. There are two networks for alignment in Figure 1(a) with nodes distinctively represented as rectangles and circles. Each one has two dense regions (highly connected sub-graphs) highlighted with different colours. For each possible pairing *(i, j) *between nodes in Figure 1 (a) of the two networks, we compute the score *S_ij _*in Figure 1(b). The scores depend on the centrality-type value as described by equation 2. Here we show that the pair of nodes *(1, a) *has the highest similarity score *S*_1*a *_= 1.60. Beginning with the seed *(1, a)*, align the neighbourhood of node {1} in *network1 *and the neighbourhood of node {a} in *network2*. The incomplete matching {*(1, a), (2, b), (3, c), (4, e), (5, f)*} emerges. Having the highest similarity score (*S*_6*g *_= 1.35) of still unaligned nodes, apparently the seed *(6, g) *should be chosen. Then seed-and-extend approaches will return the alignment result such as *Alignment1 *of Figure 1 (c). This result has aligned the whole *network1 *to only one lager region marked with green colour of the *network2*.

Analysis of the results from current seed-and-extend approaches leads us to the development of an extended seed selection strategy for GNA based multiple hub seeds. High-throughput methods such as TAP [17] identify that proteins rarely act in isolation, but rather associate with each other to perform many biological functions. Generally, protein functional modules correspond to highly connected sub-graphs in the protein interaction networks [18] and the functional modules are recorded in the interaction database as star subnetworks with a single central protein serving as a hub. Considering hubs often form functional modules in PPI networks with their neighbourhood, we can choose pairs of topological hub nodes as seeds in MHA introduced in the next section. Beginning with each hub seed, the two modules forming around the hub nodes in their corresponding networks will be aligned, MHA can obtain a more overall consistent alignment result eventually without destroying the functional modules. MHA outperforms those heuristic approaches in understanding similarities and differences between species at the systemic level.

We apply our method to align PPI networks and demonstrate that our alignment exposes far more functional complex regions than existing methods. And we can maintain the functional modules are not damaged during the heuristic alignment process by using the membership similarity of nodes to quantify to what extent the nodes can participate in functional modules associated with current seed topologically. This method is efficient in resolving the fatal problem of most conventional algorithms that the initialization selected seeds have a direct influence on the alignment result.

## Methods

### Global network alignment

A PPI network can be represented by an undirected simple network *G *= (*V_G_*, *E_G_*), where *V_G _*= (*v*_1 _, ..., *v_N_*) is a finite set of *N *vertices representing the *N *proteins, and *E_G _*is the set of edges representing the pairs of interacting proteins. Given two PPI networks *G *and *H *for various species (without loss of generality, we assume *N *<*M*, where |*V_G_*| = *N*, |*V_H_*| = *M*). The GNA problem is to find a total injective function *f *: *V_G _*→ *V_H _*which matches similar proteins and enforces as much as possible the conservation of interactions between matched pairs in the two networks. Also, no two nodes from the smaller network *G *can be aligned to the same node in the larger network *H*. To quantify how topologically similar two networks are, we can use the *edge correctness *(*EC*) measure [15]:

(1) $$EC=\frac{\left|\left\{\left(i,j\right)\in E_{G}\wedge \left(f\left(i\right),f\left(j\right)\right)\in E_{H}\right\}\right|}{\left|E_{G}\right|}*100\%$$

*EC *is the percentage of edges from a smaller network that are correctly aligned to edges in a bigger network. Naturally, when aligning two networks, we want to achieve as high *EC *as possible. GNA is NP-complete meaning as the underlying subnetwork isomorphism problem and heuristic approaches must be devised to get thus approximate solutions, especially for the large-sized PPI networks.

On the other hand maximizing the *EC *should not be the unique goal for biological networks alignment and we must strike a balance between topological and biological significances of our result. So our MHA improve the *EC *while maintaining the structure of functional modules. Although being the same as a seed-and-extend approach, MHA can solve the significant seeds chosen problem of such heuristics to a certain extent.

### The seed selection strategy and our algorithm

**Figure 2:**
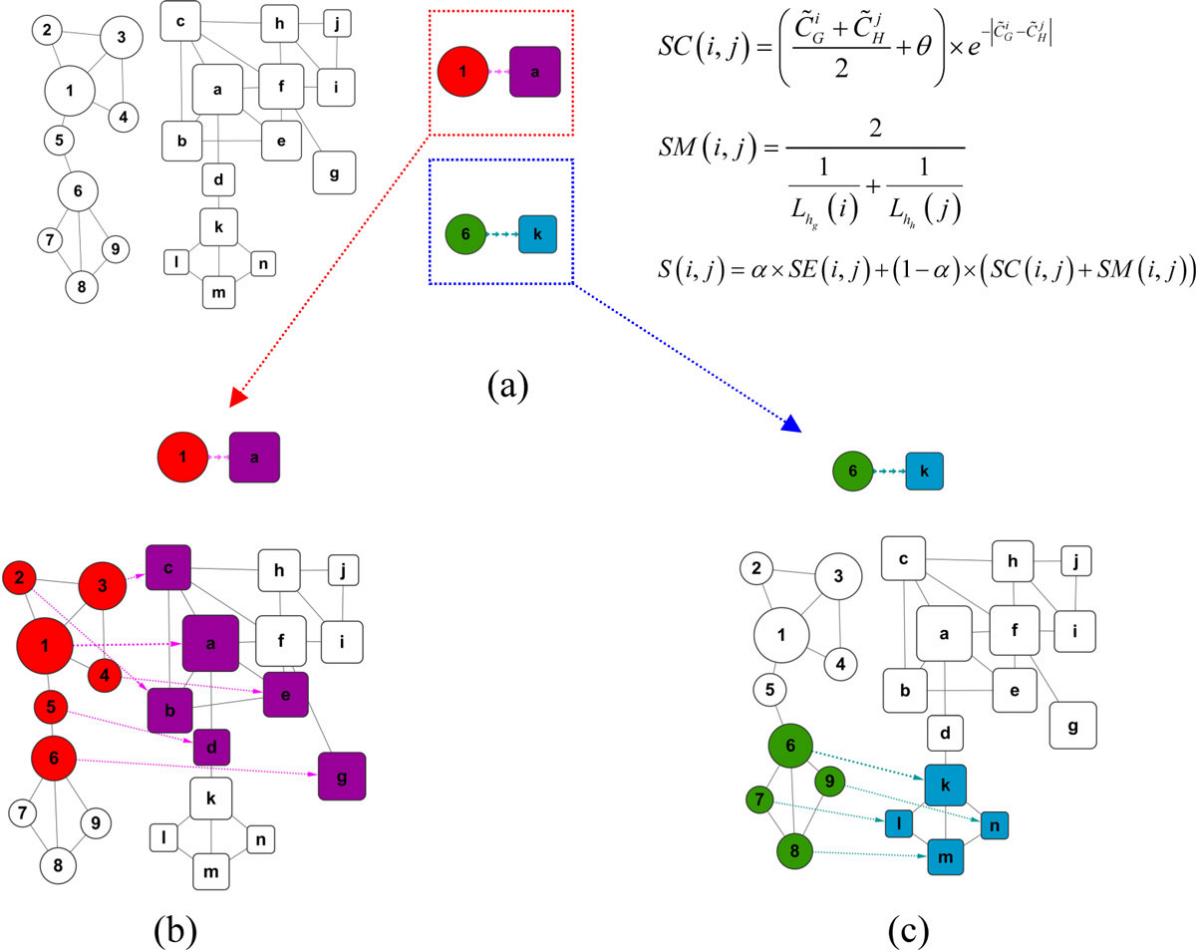
**The process of hub seeds-based alignment**. (a) Two Networks to be aligned with nodes denoted by circles and rectangles. The node significances are indicated by the sizes. The nodes with local maximum similarities are selected as hub seeds. (b) With the hub seed (1, a) the red and purple parts of networks are aligned respectively. (c) Beginning from another seed (6, k) the green and blue parts of networks are aligned.

Figure 2 shows an example of applying our method to the GNA problem. Firstly, we determine the centralities of nodes in each network and show the two networks in Figure 2(a) with the sizes of nodes proportional to their centrality values. Then the multiple hub seed set {*(1, a), (6, k)*} can be constructed for their local maximum similarities. It can be seen obviously that nodes {1, 6, a, k} are local hubs of networks respectively. Secondly, in Figure 2(b), beginning with the seed *(1, a)*, the fractional alignment result {*(1, a), (2, b), (3, c), (4, e), (5, d), (6, g)*} is obtained as shown in Figure 2(b); Simultaneity, beginning with the seed *(6, k)*, another fractional alignment result {*(6, k), (7, l), (8, m), (9, n) *} is obtained as shown in Figure 2(c). During this alignment process the dynamic membership similarity is changeable with the current aligned seed. MHA enables us to get a more overall consistent alignment shown as *Alignment2 *of Figure 1(c). MHA aligned two dense regions in *network1 *(marked with colours red and purple) according to distinct dense regions in *network2 *(marked with colours green and blue).

What follows is the detailed description of the major steps of the MHA method. In order to construct hub seeds for the seed-and-extend process, we should compute centralities and membership values of nodes in each network. An integrative network module identification and key nodes determination method family, called ModuLand [19], can be used to gain the values of the steps 1-3 straightway as follows:

Step 1: Determination of centralities of nodes in networks. NodeLand algorithm [19] iteratively determines the centrality value $C_{G}^{i}$ of node *i *∈ *V_G _*in respective networks *G *= (*V_G_*, *E_G_*). We compute the *centrality similarity (SC) *measures between all pairs of nodes (*i*, *j*) ∈ *V_G _*× *V_H _*from networks *G *and *H*. Then a *N *× *M -*sized matrix *SC *is defined as:

(2) $$SC\left(i,j\right)=\left(\frac{{\tilde{C}}_{G}^{i}+{\tilde{C}}_{H}^{j}}{2}+\theta \right)\times e^{-\left|{\tilde{C}}_{G}^{i}-{\tilde{C}}_{H}^{j}\right|}$$

(3) $$\theta =\frac{\overset{N}{\underset{i=1}{\sum }}{\tilde{C}}_{G}^{i}+\overset{M}{\underset{j=1}{\sum }}{\tilde{C}}_{H}^{j}}{N+M}$$

Respectively, ${\tilde{C}}_{G}^{i}=\text{lg}\left(C_{G}^{i}\right)$, ${\tilde{C}}_{G}^{i}=\text{lg}\left(C_{G}^{i}\right)$ and *θ *is the average centrality value of all nodes in networks *G *and *H*. There are always plentiful pairs of nodes and the differences between their centrality values are not an order of magnitude. The logarithmic value $C_{G}^{i}$ and ${\tilde{C}}_{H}^{j}$ should be employed in practice. Note that *SC*(*i*, *j*) will be large if node *i *and *j *are both significant nodes ($C_{G}^{i}$ and ${\tilde{C}}_{H}^{j}$ are all higher) in their own network and has little gap $\left(\left|{\tilde{C}}_{G}^{i}-{\tilde{C}}_{H}^{j}\right|\text{ is small}\right)$ of their centrality values. *θ *'s presence is to address the issue that a pair of similar nodes (*i*, *j*) with little gap should have a certain magnitude *SC *even if they are of very small roles in topology.

Step 2: Determination of multiple hub seeds. Here we present one concise approach suitable for the determination of hub seeds of networks. Local maxima based hub seeds is defined as: A hub seed is the pair of nodes having the locally maximal *SC *value, while all of their neighbouring nodes have lower *SC *values. The result of this step is the set *SEEDS *containing all hub seeds.

Functional modules in PPI are associated with a single central hub protein. Beginning with a hub (*h_g_*, *h_h_*) ∈ *SEEDS *to extend and align the neighbourhood of the seed, this seed-and-extend process is to construct functional modules having similar biological function and topology around the hubs respectively. For every hub seed does the same process, MHA has avoided the phenomenon that multiple functional modules in *G *are aligned to a few dense regions in *H *and can align the networks by modules.

Step 3: Determination of membership values. The membership value calculated by ProportionalHill method [19] quantifies to what extent a node can participate in a functional module associated with the current seed topologically. $L_{h_{g}}\left(i\right)$ has been used to indicate the membership value of node *i *relative to seed node *h_g _*, and a seed node gets the maximum membership value, $L_{h_{g}}\left(h_{g}\right)=1$, relative to itself. We construct *membership similarity (SM) *between all pairs of the neighbouring nodes (*i*, *j*) for a hub seed (*h_g_*, *h_h_*).

(4) $$SM\left(i,j\right)=\frac{2}{\frac{1}{L_{h_{g}}\left(i\right)}+\frac{1}{L_{h_{h}}\left(j\right)}}$$

*SM *(*i*, *j*) is the harmonic mean of $L_{h_{g}}\left(i\right)$ and $L_{h_{h}}\left(j\right)$. It is not necessary to calculate *SM *(*i*, *j*) between all pairs of nodes (*i*, *j*) ∈ *V_G _*× *V_H _*relative to all hub seeds in *H_G _*and *H_H_*. For some current seed (*h_g_*, *h_h_*), MHA only needs to get the *SM *values between neighbouring nodes around *h_g _*and *h_h _*during alignment process (in step 5) dynamically.

Step 4: Construction of the "similarity scores" matrix *S*_*N*×*M*_. We implement MHA by using the topological similarity (*SC*) between nodes in two networks, along with the sequence similarity (*SE*) given by the BLAST [20] E-value score between protein sequences. BLAST E-values are a standard measure for deciding whether two proteins are orthologs. Note that the "perfect" alignment should minimize *centrality*, *membership *and *sequence *differences between nodes. Hence, the *similarity *score between nodes *i *and *j*, *S*(*i*, *j*), is computed as follows:

(5) S(i,j)=α×SE(i,j)+(1-α)×(SC(i,j)+SM(i,j))

*S *is *N *× *M *-sized matrix and *S(i*, *j*) is an kind of dynamic similarity. Different seed considered among the alignment process leads to different *SM *(*i*, *j*) and S(*i*, *j*). The weight *α *can be adjusted to assign relative importance to biological and topological data, depending upon the confidence level attributed to them and the type of results sought. In our implementation we assign the weight (*α *= 0.6). This parameter has been discussed in the following section.

Then we present the detailed description of MHA based on the matrix *S*, and in the following part we define the specific concepts used.

Algorithm *MHA (G*, *H)*

Construct the matrices *SC*, *SE *and the set *SEEDS*.

Initialize alignment *A *to an empty set and alignment score vector *B *equals to 0.

**for a **hub seed (*h_g_*, *h_h_*) ∈ *SEEDS ***do**

   Add (*h_g_*, *h_h _*) to alignment *A*, *B*(*h_g _*, *h_h_*) = *S*(*h_g _*, *h_h_*).

   **for all ***k *∈ **{**1, ..., *D*} **do**

      Construct a bipartite graph $BP\left(N_{G}^{k}\left(h_{g}\right),N_{H}^{k}\left(h_{h}\right),E\right)$

      Compute *SM *(*i*, *j*) relative to the seed and assign the weight *ω*(*i*, *j*) = *S *(*i*, *j*).

      Solve the *Maximum Weight Bipartite Matching Problem *by the Hungarian algorithm [16].

      To each optimal matching (*u*, *v) *found above, if and only if *S *(*u*, *v*) >*B*(*u*), then add (*u*, *v*) to the current alignment *A*, *B*(*u*) = *S*(*u*, *v*).

   **end for**

end for

**return **alignment *A*.

The *k^th ^*neighbourhood of node *h_g _*in network *G*, $N_{G}^{k}\left(h_{g}\right)$, is defined as the set of nodes of *G *that are at distance ≥ *k *from *h_g _*. Hence, $N_{G}^{k}\left(h_{g}\right)$ is the *k^th ^*still unaligned neighbourhood and can be thought of as the "ball" of nodes around *h_g _*up to and including nodes at distance *k*. This allows us to model insertions and deletions of nodes in the paths conserved between two networks. *D *is the longest distance restricted.

A bipartite graph, *BP *(*V*_1_, *V*_2_, *E*), is a graph with a node set *V *consisting of two partitions, *V *= *V*_1 _∪ *V*_2_, so that every edge *e *∈ *E *connects a node from *V*_1 _with a node from *V*_2 _; that is, there are no edges between nodes of *V*_1 _and there are no edges between nodes of *V*_2 _-- all the edges "go across" the node partition.

The set *A *contains pairs of nodes which are the optimal matching results during the process of MHA. The matrix *B *records the alignment score when a matching is added to *A*. While using the multiple hub seeds, the object node in network *H *to which MHA has matched a node in *G *can be changeable. Membership values are related to the current aligned seeds. Beginning with different seeds, we will get different similarity score influenced by membership values, then a node in *G *should be aligned to the node that they can get the highest similarity score together. The significance of dynamical and changeable membership value is that a node can select the best associated hub seed to form a functional module, and also several smaller modules in one network will never be covered constrainedly by a larger one in another network along with the alignment process.

### Computational complexity of MHA

NodeLand [19] algorithm for determination of centralities of nodes is structurally similar to a breadth-first search, therefore the worst-case runtime complexity is *O*(N*(*N *+ |*E*|)), respectively, where *N *is the number of nodes and |*E*| is the number of edges in the network. The presented ProportionalHill [19] method has a runtime complexity of *O*(d*|*E*|*h), with *d *being the average node degree and *h *being the number of the identified hubs. Solving the alignment for bipartite graph *BP *(*V*_1_, *V*_2_, *E*) takes *O*((|*V*_1_|+|*V*_2_|)*(|*E*|+(|*V*_1_|+|*V*_2_|)*log((|*V*_1_|+|*V*_2_|)))). Therefore, the total time complexity of MHA algorithm for aligning networks *G *= (*V_G_*, *E_G_*) and *H *= (*V_H_*, *E_H_*) is smaller than *O*(h*|*V_G_*|*(|*E_G_*|+|*V_G_*|*log(|*V_G_*|))) and the space complexity is *O*(|*V_G_*|*|*V_H_*|+|*E_G_*|+|*E_H_*|) clearly.

## Results and discussion

### Data sets and summarized statistics for our seed selection strategy

We implement the proposed MHA in the C programming language and use it to identify the common alignment graph between PPI networks including the S. cerevisiae (S), D. melanogaster (D), H. sapiens (H) and M. musculus (M). The interaction data is available in October 2008 release of the DIP [21] molecular interaction databases (DIP: http://dip.doe-mbi.ucla.edu/). The statistics for the PPI networks are shown in table 1. GO enrichment measures the components in an identified alignment with respect to the biological process annotation of GO, for each species separately. We use the tool GO TermFinder [23] to compute components functional enrichment P-values.

**Table 1:** Description of aligned PPI networks

Species	Vertex	Edge
S. cerevisiae (S)	4963	17570
H. sapiens (H)	1607	1951
M. musculus (M)	599	513
D. melanogaster (D)	7498	22864

**Figure 3:**
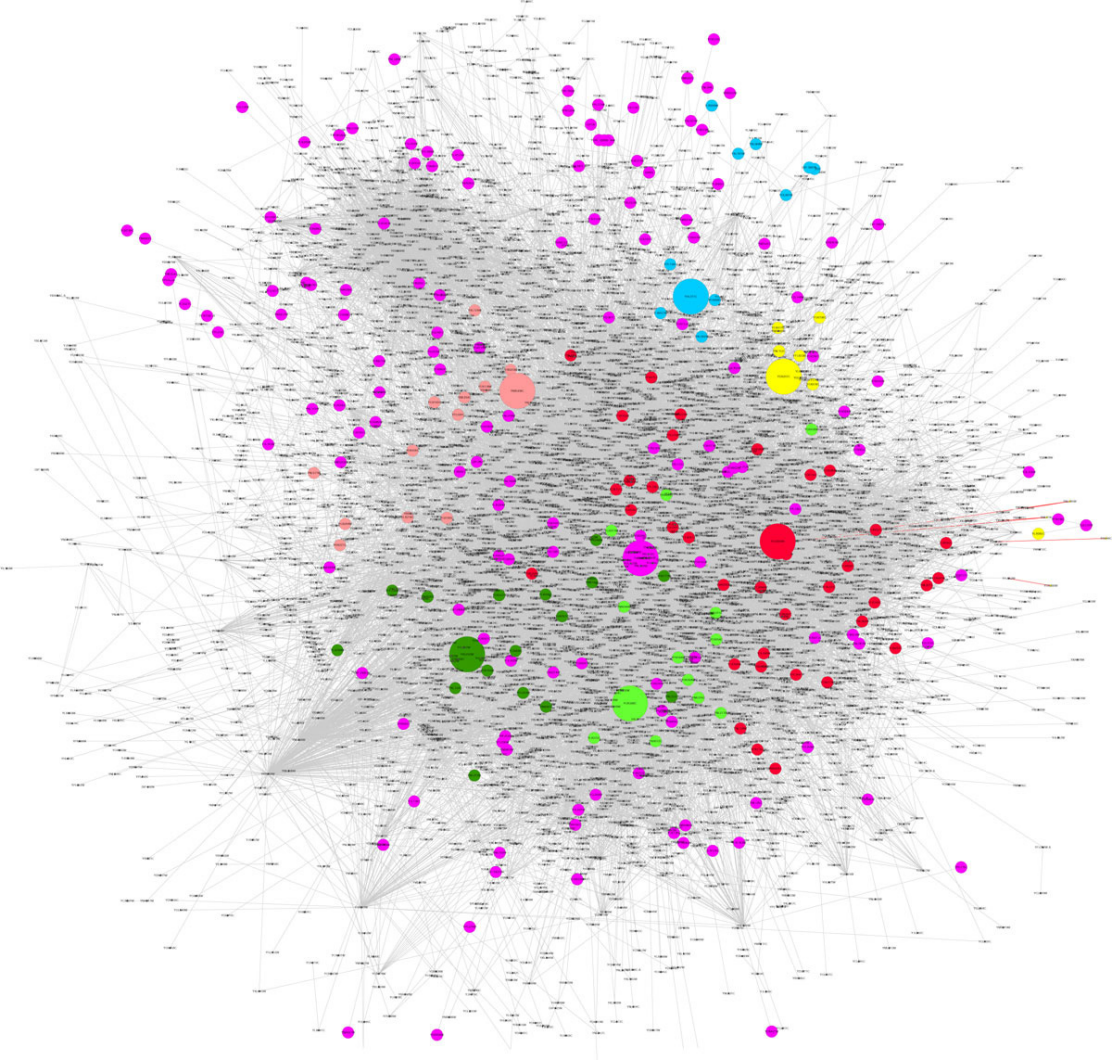
**Some functional modules formed around seeds in yeast**. From a large-size seed some nodes painted same colour will be aligned. And the same colour nodes will form a functional module around the seed node in our method.

For each pair of networks table 2 shows our alignment results. When we align from the S. cerevisiae (S) to D. melanogaster (D) PPI networks, the set *SEEDS *consisting of 255 hub seeds is constructed. And there are 106 modules with obvious biological significance in yeast and 45 functional modules in fly formed around the seeds after the alignment process eventually. Performing our method for aligning M. musculus to H. sapiens PPI networks, the set *SEEDS *consisting of 91 hub seeds is obtained. There are 55 functional modules in mouse and 45 functional modules in human formed around the seeds after the alignment process eventually. All these functional modules have obvious biological process annotation of GO. The conserved modules shown in table 2 are those who execute the same functions in their respective networks and have the same annotation of GO terms in our alignment results. Conserved edge is the percentage of edges from a smaller network that are correctly aligned to edges in a bigger network. These results show that we can maintain the functional modules undamaged during the heuristic alignment process and align the networks by modules. Simultaneously our method is efficient in achieving a high percentage of conserved edge. In Figure 3, we give some functional modules of yeast that formed during our alignment process from yeast to fly PPI network. From a large-size seed some nodes painted same colour will be aligned. And the same colour nodes will form a functional module around the seed node.

**Table 2:** Statistic analysis of maintained functional modules in our result

	Hub seeds	Conserved edge	Functional modules of first species	Functional modules of second species	Conserved modules
S to D	255	2649(15.91%)	106(42%)	45(18%)	8(17.78%)
H to S	175	515(26.40%)	76(43%)	51(29%)	14(27.45%)
M to S	57	134(26.12%)	40(70%)	18(32%)	9(50%)
M to H	91	226(44.15%)	55(60%)	45(49%)	17(37.78%)
H to D	264	476(24.42%)	116(44%)	27(10%)	7(25.93%)

**Figure 4:**
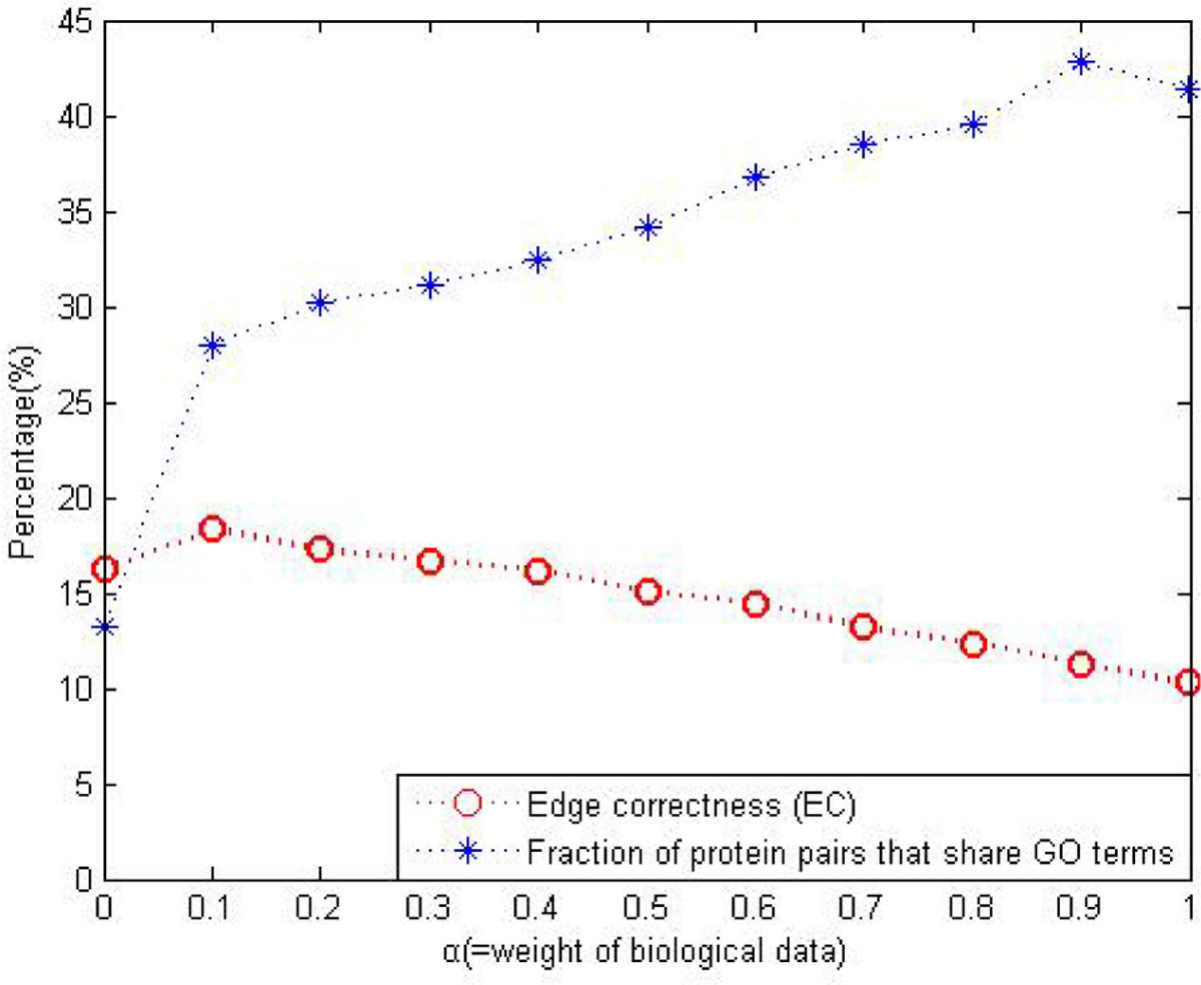
**Effect of the parameter *α***.

The best currently published network alignment algorithm, MI-GRAAL, uses a cost function relying on a highly constraining measure of topological similarity and achieved higher percentage of conserved edge (usually at about 50%) than our results. But the only one object for MI-GRAAL is to optimize the *EC *no fear of destroying the functional modules in original networks. For proteins always associate with each other to perform biological functions, it is more important to maintain the functional modules unchanged rather than maximize the *EC *value. As shown in Figure 4 we can improve our *EC *values shown in table 2 simply by decreasing the parameter *α*. And also our strategy of seed selection can be used as the reference and complement of other heuristic methods to seek more meaningful alignment results.

### Enriched functional modules of alignment network

**Figure 5:**
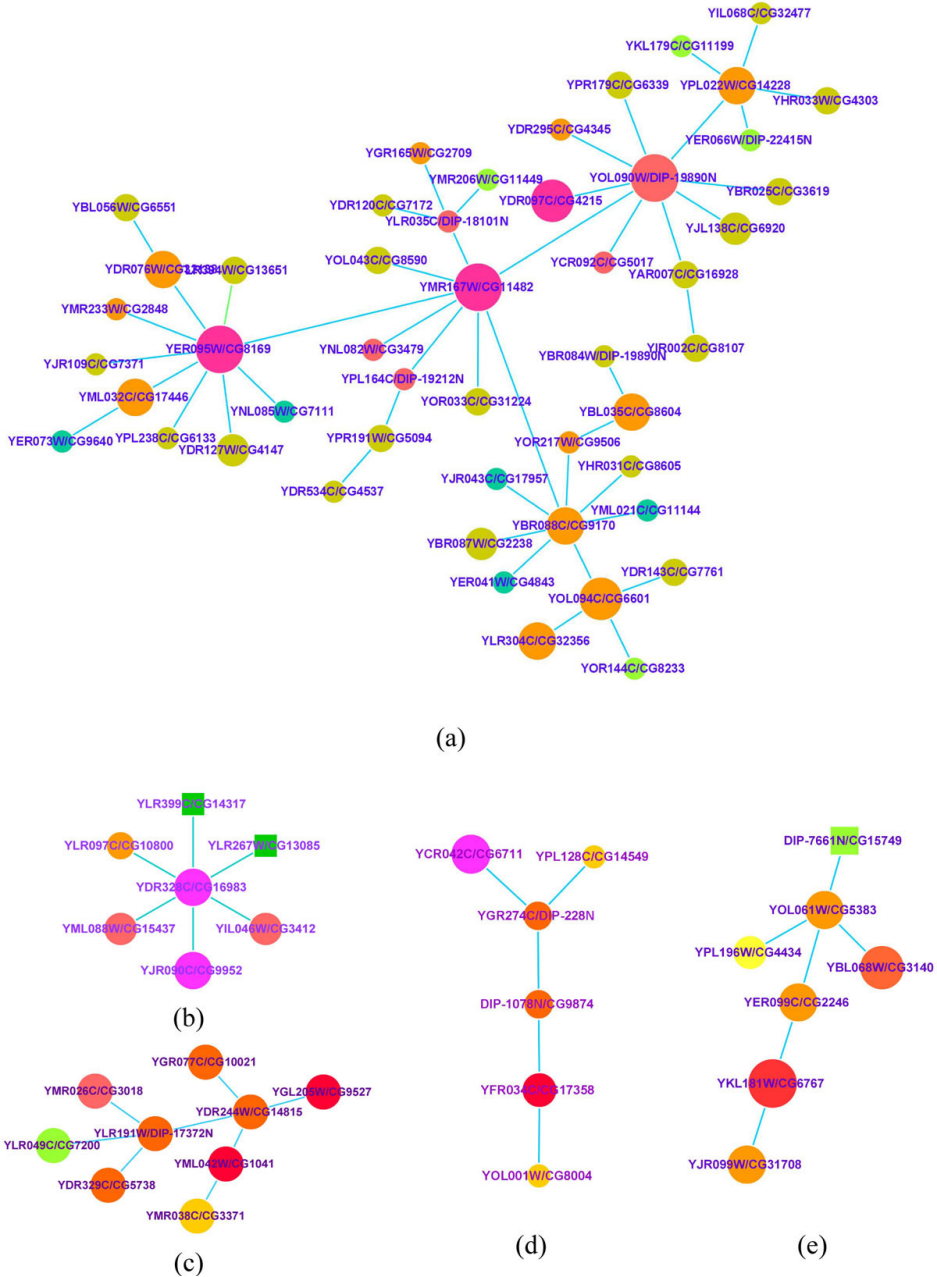
**Some functional subnetworks of our result**. The node labels indicate the corresponding "yeast/fly" proteins. (a) The largest connected component of the yeast-fly GNA.

As a typical case study we use MHA to identify the common alignment network between PPI networks the S. cerevisiae (yeast) and D. melanogaster (fly). We construct the set *SEEDS *consisting of 255 hub seeds. The common graph corresponding to the global alignment between the yeast and fly PPI networks is comprised of 2649 edges with edge correctness of 15.08% (*α *= 0.6). The alignment graph consists of many disconnected components, with the largest component having 48 edges amongst 49 proteins (in Figure 5(a)). Table 3 presents the results of our algorithm and IsoRank [14], in terms of conserved interactions, size of the largest component and P-value. We know that MHA produces a more optimal solution, which can be found at no additional computational cost. Retrieving in particular 86% more conserved interactions than IsoRank for a given level of sequence similarity between two networks denotes that our alignment result is more topologically similar. Also, its largest component with P-value of 5.83E-13 has a significant function of DNA binding achieved congruously in both yeast and fly (in table 4). In contrast the function of IsoRank algorithm's largest component is unnoticed with P-value of 0.00098. As shown in Figure 5, the components discovered simultaneously span various topologies, from linear pathways (Figure 5(d), Figure 5(e)) to components corresponding to protein complexes (Figure 5(a), Figure 5(b) and Figure 5(c)). Each one has coherent function (identified by same GO term) in the two species. The detailed GO terms and P-value of these components are presented in table 4. Finally, we give some components having functionality only in yeast or fly detected by MHA in table 5. The most significant module with P-value of 4.30E-21, comprised of 49 aligned proteins, is obtained by our alignment in yeast PPI.

**Table 3:** Comparison with IsoRank

*Algorithm*	*IsoRank *	*MHA *
*Number of conserved interactions*	1420	2649
*Size of the largest component*	35	48
*P-value*	0.00098	5.83E-13

**Table 4:** Enriched functions of alignment network

Figure		GO term	P-value
Figure 4(a)	Yeast	structure-specific DNA binding	5.83E-13
	Fly	mismatched DNA binding	1.27E-11
Figure 4(b)	Yeast	SCF ubiquitin ligase complex	1.33E-11
	Fly	SCF ubiquitin ligase complex	1.05E-10
Figure 4(c)	Yeast	peroxisome	4.71E-12
	Fly	peroxisome	0.00385
Figure 4(d)	Yeast	transcription factor TFIID complex	1.71E-06
	Fly	transcription factor TFIID complex	0.00292
Figure 4(e)	Yeast	ribose phosphate diphosphokinase activity	3.18E-11
	Fly	ribose phosphate diphosphokinase activity	1.62E-06

**Table 5:** Prediction of functional modules

Species	GO term	Cluster frequency	P-value
Yeast	DNA repair	25 of 49 genes	4.30E-21
Yeast	organellar small ribosomal subunit	7 of 8 genes	6.25E-15
Yeast	90S preribosome	9 of 12 genes	2.51E-14
Yeast	proteasome complex	8 of 14 genes	1.53E-13
Fly	Hedgehog signaling complex	4 of 12 genes	4.61E-12
Fly	SCF ubiquitin ligase complex	4 of 7 genes	1.05E-10
Fly	cullin-RING ubiquitin ligase complex	4 of 7 genes	8.25E-10

We emphasize that our components are rich in biological functions notwithstanding they are just subnetworks of the sparse alignment graph. Enrichment functions in our alignment graph profit from the selection of initialization multiple hubs. Around these hubs neighbourhood proteins form consistent and conserved functional modules in two PPI networks simultaneously. Our alignment result is more biologically similar.

### Identification of functional orthologs

Proteins that are aligned together in the global alignment should have similar interaction patterns in their respective species and are thus likely to be functional orthologs [3]. Recently, there has been a lot of interest in the discovery of functional orthologs (FO). In particular, Bandyopadhyay et al. [3] took a fairly complex approach to FO detection between yeast and fly through LNA, and the probability of each short-listed pair of proteins being true FOs is computed. We apply MHA to the detection of functional orthologs (i.e., sets of proteins that perform the same function in two or more species), and compare of our results with Bandyopadhyay et al.'s results [3]. Our method has 162 pairs of proteins consistent with their results and, moreover, often resolves the ambiguity in their predictions. Some examples of our predicted FO matching Bandyopadhyay et al.'s predicted FO are shown in table 6. For each predicted functional ortholog the Number of Conserved interactions (NC), the Number of interactions in Yeast (NY), the Number of interactions in Fly (NF) and the probability of the FO being true are exposed.

**Table 6:** Comparison of our results with Bandyopadhyay et al.'s results [3].

Yeast/Fly pair	(NC, NY, NF)	**Prob**.
YBR109C/CG17769	(6,61,26)	44.39%
YDR244W/CG14815	(6,7,36)	100.00%
YLR447C/CG2934	(5,89,18)	40.33%
YLR026C/CG1467	(5,19,27)	0.00%
YKL067W/CG18584	(4,8,51)	0.00%
YDL081C/CG4087	(4,9,44)	50.51%
YER081W/CG6287	(4,83,17)	40.34%
YER112W/CG31990	(4,41,7)	100.00%
YBL050W/CG6625	(4,36,4)	100.00%
...	...	...

### Effect of the parameter *α*

To measure the biological quality of the alignment result, we count the fraction of aligned pairs that have at least 1 GO term [22] in common, estimated by blue curve in Figure 4. Simultaneously we compute the edge correctness (EC) to evaluate the topological quality of the alignment result, marked by red curve. When choosing the most appropriate value of the free parameter *α*, we rejected the choice corresponding to the largest common alignment graph size (the largest EC); thus, the *α *leading to the largest-size alignment graph may not be a biologically appropriate choice. Instead, for the choice of *α*, we compared the biological quality with the topological quality and chose the *α *= 0.6 that is used to adjust the assignment of the relative importance to the topological and biological data.

### The error tolerance of MHA

**Figure 6:**
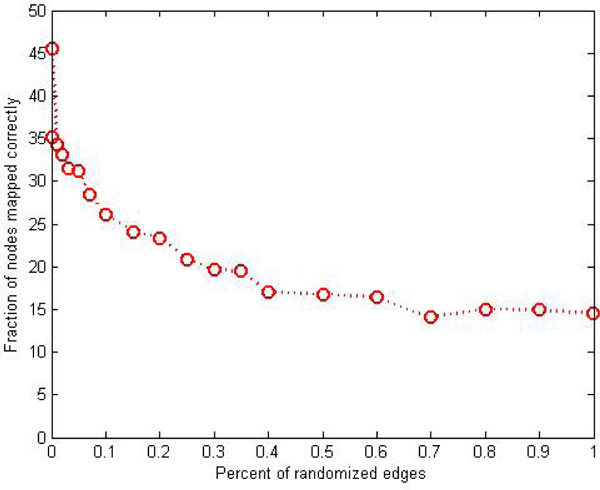
**Effect of error on algorithm's performance**.

Our simulations indicate that the algorithm is tolerant to errors in the input networks (Figure 6); this is valuable since PPI networks have high false positive and false negative rates. To evaluate the algorithm's error-tolerance we randomized a fraction of its edges using the Maslov-Sneppen trick that preserves node degrees [24]: we randomly choose two edges *(a, b) *and *(c, d)*, remove them, and introduce new edges *(a, d) *and *(c, b)*. For each choice of the percentage of randomized edges, we compute the fraction of nodes that are mapped to exactly same objects in the original graph and randomized graph after GNA. Clearly, the algorithm's performance degrades smoothly and very slowly. These simulations suggest our results are quite robust to errors in PPI data.

## Conclusions

In this paper, the focus is on the GNA problem, and an intuitive yet powerful algorithm is proposed for computing the global alignment of two PPI networks; Unlike previous heuristic algorithms whose initialization seeds have a very deep influence on the alignment result, MHA, simultaneously, using multiple hub seeds and dynamic membership value, enable us to get a more overall consistent alignment and has avoided the phenomenon that multiple functional modules in one network are aligned to a few dense regions in another. Matching biological networks of different species is expected to be a valuable tool, since the results can be used to knowledge transfer between species, detection of new conserved pathways and prediction of PPI data. We expect the methods proposed in this paper to have a direct impact on these applications and become a remedy of other heuristic algorithms.

## Competing interests

The authors declare that they have no competing interests.

## Authors' contributions

BW and LG designed the study. BW implemented the method, performed the experiments and analyzed the data. All authors contributed to discussions on the method. BW and LG wrote the manuscript. All authors revised the manuscript and approved the final version.
